# Older Adults’ Exposure to Food Media Induced Unhealthy Eating during the COVID-19 Omicron Lockdown? Exploring Negative Emotions and Associated Literacy and Efficacy on Shanghainese

**DOI:** 10.3390/foods13121797

**Published:** 2024-06-07

**Authors:** Wen Jiao

**Affiliations:** School of Communication, Soochow University, Suzhou 215123, China; wjiao@suda.edu.cn

**Keywords:** food media, cognition, negative emotions, food literacy, health consciousness, eating self-efficacy, older adults, unhealthy eating

## Abstract

The COVID-19 pandemic, propelled by the highly transmissible Omicron variant, had a global impact and significantly affected Shanghai, a major city in China. This study investigates how food media exposure influenced unhealthy eating habits among older adults during the COVID-19 lockdown in Shanghai, focusing on the roles of negative emotions, food literacy, health consciousness, and eating self-efficacy. The random sample comprised 400 individuals aged ≥50 years who lived in Shanghai from March to June 2022. A path and correlation analysis was performed. The exposure of older adults to food media resulted in the acceleration of unhealthy eating. The relationship was significantly exacerbated by food literacy and negative emotions. In contrast, eating self-efficacy and health consciousness effectively countered the media. The enhanced pathway from food-related media influence to eating habits through negative emotions or self-efficacy towards health awareness and food literacy showed significant effects. The findings provide insights for future research and public health strategies. Importantly, this study has practical significance for media professionals, public health decision-makers, and healthy food businesses regarding how to enhance older adults’ cognition to respond to unhealthy eating crises.

## 1. Introduction

An outbreak of the highly infectious Omicron variant of the coronavirus disease (COVID-19) emerged in Shanghai, the largest city in China, in March 2022 [1]. This ranks among the most severe since the initial report in Wuhan in 2020. The pandemic has shifted people‘s dietary habits, which was becoming a public health issue due to changes in social interactions owing to restricted access to daily grocery shopping and encouragement of cooking at home rather than dining out [2]. An unhealthy diet not only leads to malnutrition but also affects the immune system and increases the risk of noncommunicable diseases [3]. Several studies on self-reported eating habits suggest that individuals’ appetites have changed, partly because of pandemic-associated negative emotions, such as anxiety, fear, and distress [4,5]. Furthermore, the consumption of unhealthy foods, such as high-calorie fatty sweets/snacks between meals, has increased [6,7].

Older adults are especially susceptible to the adverse consequences of a COVID-19 lockdown owing to age-related physiological changes and a greater prevalence of underlying health conditions [8]. Those groups of individuals encounter both physical and psychological hardship, including the deterioration of taste and smell [9], isolation [10], and the loss of loved ones [11]. In such circumstances, locating and savoring delicious, unhealthy foods may provide them with some mental solace. Unhealthy foods in particular tend to have a more intense taste and flavor, and this stimulation can be more pleasurable and satisfying for older adults. This, in turn, worsens the overall health risks. Owing to pandemic-related restrictions, older individuals confined to their homes rely heavily on media exposure as their main method of communication with the outside world. Studies regarding the impact of media exposure on unhealthy eating behaviors and cognitive processes in older individuals are limited, and the present study represents the first attempt in this area.

This study aimed to fill this gap by addressing how older people learned or acquired unhealthy eating behaviors from exposure to food messages in media during the COVID-19 Omicron lockdown in Shanghai, China. This study contributes to a more detailed understanding of the cognitive mechanisms of unhealthy eating behaviors by focusing on the triadic interactions between food media, individual agency, and environmental factors. This process was not a direct influence of the media on individual behavior; rather, the relationship was indirect and influenced by several psychosocial determinants as the COVID-19 lockdown progressed, including negative emotions, food literacy, health consciousness, and eating self-efficacy. This study also adds to the growing body of literature on pandemic preparedness and response strategies.

## 2. Literature Review

### 2.1. Food-Evoked Negative Emotions

The media’s portrayal of food has the potential to affect people’s thoughts and emotions regarding food, as well as how they ultimately choose to spend their food budgets [12]. Although little is known about exposure to food media messages and their consequences, food media exposure may evoke different types of negative emotions, such as fear, depression, and anxiety [13,14]. For instance, food media overemphasize food safety concerns during the COVID-19 outbreak, including the risk of food contamination or virus transmission [15]. Such unfavorable reporting may evoke people’s distrust and negative feelings towards food [16].

Negative emotions can aggravate unhealthy cravings, leading people to embrace “comfort food,” such as energy-dense and flavorful foods, whose frequent consumption is not conducive to a healthy lifestyle [17]. Negative feelings, such as psychological distress, were accompanied by uncontrollable emotional eating behaviors in populations experiencing lockdowns due to COVID-19 outbreaks, including 485 individuals aged 12–75 years in China [18] and 136 adults in the United Kingdom [19]. Because of overeating, consumption of unhealthy food, such as high-calorie fatty sweets/snacks, has been observed in several studies [20,21].

Negative emotions such as fear and anxiety, exacerbated by media exposure during the pandemic, can lead to unhealthy eating behaviors. Studies show a positive correlation between media-induced negative emotions and the consumption of comfort foods. Based on this knowledge, the following two hypotheses are proposed:

**H1:**  *Generally, food media exposure was positively associated with unhealthy eating.*

**H2:**  *During the COVID-19 lockdown, negative emotions positively mediated the relationship between food media exposure and unhealthy eating.*

### 2.2. Food Literacy

“Food literacy” is the accumulation of interconnected knowledge, mindsets, and behavioral patterns concerning food planning, selection, preparation, and consumption [22]. Increased food literacy correlates with greater self-control and impulse control, with highly food-literate individuals being more inclined to make informed decisions regarding balanced and diverse nutrition [23]. Many studies have supported the idea that food literacy is a key mediator between media exposure and healthy eating habits; however, discussions on the significance of food literacy in relation to unhealthy dietary habits are limited.

Steils and Obaidalahe [24] asserted that the rapid spread of food-related content facilitated by social media could be used as a teaching tool and policy measure to boost food literacy and support healthy eating habits. Melki et al. [25] showed that perceived knowledge positively reinforced the impact of media exposure and health-related behaviors among adults living in Lebanon throughout the initial phase of the COVID-19 pandemic in spring 2020. Given that individuals do not entirely abstain from consuming unhealthy foods, it is important to investigate how food literacy can influence the relationship between media exposure and unhealthy dietary preferences.

With this in mind, one major goal of this study is to investigate how food literacy may mediate the connection between media exposure and poor eating habits within the context of the COVID-19 lockdown. Thus, the following hypothesis is proposed:

**H3:**  *During the COVID-19 lockdown, food literacy positively mediated the relationship between food media exposure and unhealthy eating.*

### 2.3. Health Consciousness

A person’s “health consciousness” reflects their level of interest in maintaining a healthy lifestyle [26]. Several studies concluded that media information on health and nutrition empowers health consciousness. Populations such as patients in health care [27] and women of childbearing age [28] use media as a support tool for learning health-related content as an effective way to improve health awareness. In turn, people who valued their health were more likely to seek health-related messages, discuss what they had learned from media sources, and ultimately apply what they had learned [29]. Therefore, this study infers that food media exposure positively affects health consciousness in a similar manner.

The influence of health consciousness on poor and unfavorable diets has been studied extensively in recent years. According to research by Divine and Lepisto [30], health-conscious people in the US tend to favor white meat, fruits, and vegetables, while avoiding the unhealthy options of red meat, snacks, and sugary drinks. Hartmann et al. [31] found that men who were less health conscious consumed more meat, sweetened beverages, alcoholic beverages, and convenience foods than women; this behavior increased their risk of obesity. Considering this evidence, the present study argues that being health conscious had an adverse impact on the choice of an unhealthy diet during the COVID-19 lockdown.

Past evidence supports the idea that food media exposure positively affects health consciousness and that health consciousness inhibits unhealthy eating. This study infers that health consciousness is a key mediator of the negative connection between the two as reflected in the following hypothesis:

**H4:**  *During the COVID-19 lockdown, health consciousness negatively mediated the relationship between food media exposure and unhealthy eating.*

### 2.4. Eating Self-Efficacy

People’s confidence in their own abilities to carry out desired actions in a given context is often referred to as their “self-efficacy” [32]. Prior studies have established that self-efficacy can help regulate unhealthful behaviors. Fitzgerald et al. [33] identified dietary choices as either healthy or unhealthy food patterns by asking Irish adolescents about the frequency of various food groups in their diet and showed that self-efficacy positively predicted healthy nutritional choices and negatively predicted unhealthy nutritional choices. Churchill et al. [34] showed that individuals with low confidence in their ability to control their diet were especially susceptible to unhealthy eating habits, such as consuming high-calorie snacks. Diotaiuti et al. [35] further demonstrated the impact of cognitive appraisals on perceived self-efficacy and distress during the COVID-19 lockdown, providing empirical support for the critical role of self-efficacy in managing distress and health-related behaviors; the study underscores the importance of self-efficacy in regulating dietary behaviors and managing psychological distress during public health crises. Thus, the existing literature suggests that eating self-efficacy is a focal construct for predicting unhealthy eating patterns. The following hypothesis is proposed:

**H5:**  *During the COVID-19 lockdown, eating self-efficacy negatively mediated the relationship between food media exposure and unhealthy eating.*

### 2.5. Potential Interaction

Little is known about how negative emotions, food literacy, health consciousness, and eating self-efficacy interact with the cognitive processes of food media exposure and unhealthy eating habits. According to a protective mechanism suggested by van Kampen [36], the negative feelings brought on by the COVID-19 lockdown may play an essential yet indirect role in producing behavioral reactions to unhealthy eating patterns through health consciousness or food literacy. Positive cognition might be activated in reaction to impending hazards and induce an arousal response in rational awareness to eliminate the negative feelings of a mismatch between reality and one’s expectations [37].

Researchers have studied the impact of self-efficacy on valued outcomes in many fields, including health consciousness and food literacy. Despite the paucity of research, eating self-efficacy may positively influence health outcomes. For instance, Anderson et al. [38] analyzed the eating habits of health conscious African-American consumers and found that their actions were contingent on their belief in their ability to identify and select nutrient-dense options. Nutritional self-efficacy and health awareness were found to be positively correlated [39], and both were important for health-related characteristics when making food choices [40,41]. Based on these reasonings, one research question is proposed:

**RQ1**: How do negative emotions, eating self-efficacy, health consciousness, and food literacy interact in the relationship between food media exposure and unhealthy eating during the COVID-19 lockdown?

## 3. Materials and Methods

### 3.1. Research Design

This study was an extension of the Corona Cooking Survey (CCS), which involved the collaboration of researchers worldwide [42,43]. The original CCS was a two-wave cross-sectional study that examined how people’s food-related knowledge, abilities, and behaviors were affected by the COVID-19 pandemic, as well as the various individual and environmental factors that contributed to these effects. Thus, individual responses can be identified in terms of food media influence, food buying, cooking, and eating habits since the onset of the COVID-19 pandemic.

A web-based survey was conducted among Shanghai residents aged 50 or older, approved by the University of Macau Ethics Committee (approval code: SSHRE22-APP018-FSS; approval date: 13 May 2022). The questionnaire consisted of 20 questions in seven parts: food media use, current feelings about the COVID-19 lockdown, health consciousness, food consumption, body satisfaction, self-efficacy in picking the right food, and basic information. Two screening questions were set to obtain participation permission and to ensure that residents lived in Shanghai during the March–June 2022 lockdown.

The first-tier city of Shanghai was chosen as the sampling location because a new round of COVID-19 Omicron with a high infection rate erupted there around March 1, 2022 [44]. After a two-month unprecedented “area-separated control” lockdown period, Shanghai began easing COVID-19 restrictions on 1 June 2022 [1]. Therefore, it is of significant value to explore Shanghai residents’ dietary behavior with typical and time-sensitive representativeness. The outbreak in the city was a sufficient reason to conduct a study focused on its residents.

### 3.2. Procedure

Qualtrics XM platform was contracted to distribute the questionnaire through simple random sampling from July to August 2022, targeting residents of Shanghai aged 50 years or older. In mainland China, the retirement age for most individuals is 50 [45]. At this age, individuals may still appear youthful, but their physical abilities are declining [46]. This study aligned with previous studies by using 50 years of age as the benchmark for defining older adults [47,48,49]. The Qualtrics administrators sent email invitations to potential participants through authorized partner companies in China, informing them that the survey was strictly for research purposes. Completing the survey required 10–20 min and included an incentive offer, with compensation options, such as gift cards or vouchers for the respondents.

A total of 1389 individuals participated in the survey. However, exclusions were made based on specific criteria: 189 respondents (13.6%) did not reside in Shanghai in 2022, 167 respondents (12.0%) were under the age of 50 years, 16 respondents (1.2%) completed the survey faster than the median time, and 47 respondents (3.4%) who only partially completed the questionnaires were excluded from the analysis. Consequently, 462 respondents (33.3%) fully completed every survey question as required. After data cleansing, the final analysis included 400 valid respondents who were living in Shanghai and were aged ≥50 years.

### 3.3. Measurements

The measurement of food media exposure reflected how often respondents encountered or used media messages about food and nutrition from 12 sources [12], namely cookbooks, other print media, Sina Weibo, television shows, Facebook, forum websites, Instagram, WeChat, YouTube, TikTok, Bilibili, and other social media. The respondents rated the frequency with which they used each source on a scale of 1 (never) to 5 (all the time). A higher average score indicated higher food media exposure.

Unhealthy eating was measured using ten questions about the intake of at least one portion of the following food products: processed meat, red meat, sweet snacks, salty snacks, sweetened beverages, alcoholic beverages, fast food, animal fats, coconut oil, and white grains [5,7]. Each item was rated from 1 (less than a few times per month or never) to 5 (more or many times a day). The higher the average score, the higher the level of poor eating quality.

Negative emotions were measured using 13 items in two dimensions drawn from earlier studies on psychological distress [43] and fear [14]. Respondents rated each psychometric property in terms of their agreement with statements, ranging from 1 (low agreement) to 5 (high agreement) (Cronbach’s alpha = 0.834 for psychological distress and 0.888 for fear). Negative emotions regarding COVID-19 were computed by dividing the average score of the two dimensions by two. A higher mean score indicated a more severe degree of negative emotional distress.

Food literacy was measured using 11 questions in three dimensions asking respondents how often they had performed cooking-related actions pertaining to the planning, selection, and preparation of food during the COVID-19 lockdown [42,43]. Each item was rated from 1 (never) to 5 (every time) (Cronbach’s alpha = 0.773 for planning, 0.702 for selection, and 0.731 for preparation). Finally, food literacy was computed by dividing the sum of the average scores of the three dimensions by three. A greater change in food literacy during the COVID-19 lockdown was indicated by a higher mean score.

Health consciousness, which refers to how often people were concerned about their health during the COVID-19 lockdown, was measured using nine questions [26]. Respondents rated their frequency of concern for each item on a scale of 1 (never) to 5 (all the time) (Cronbach’s alpha = 0.870). When asked about their health consciousness during the COVID-19 lockdown, those with higher average scores reported more positive internal states of mind.

Eating self-efficacy was measured using 11 questions asking respondents about their confidence in healthy eating and their capacity to differentiate between healthy and unhealthy eating [50,51]. Each item was rated on a Likert scale ranging from 1 (strongly disagree) to 5 (strongly agree) (Cronbach’s alpha = 0.858). A higher mean score indicated a greater degree of eating self-efficacy.

Other demographic information used as control variables included sex, age, education, income, employment status, general financial struggles, financial struggles in purchasing food, and body mass index [5,42]. Detailed information regarding these measurements is provided in the Supplementary Materials.

### 3.4. Data Processing

SPSS Statistics 28 was used for data analysis. A sociodemographic analysis was performed to characterize the Chinese sample from July to August 2022. Additional analyses were performed on the means (M), standard deviations (SDs), and zero-order correlations (r) of primary variables. Subsequently, path analysis was conducted using AMOS 28 to validate the proposed model. The maximum likelihood discrepancy method, with a bootstrap sample of 5000, one random seed, and 95% bias-correlated confidence intervals (CIs) [52], was used to examine the different types of indirect effects, overall saturation, and goodness-of-fit indicators. Indirect effects were considered significant if CI limits were either both positive or both negative [53].

## 4. Results

### 4.1. Descriptive Statistics

We analyzed 400 validated and complete survey responses. Respondents, who all resided during the COVID-19 lockdown in 2022 in Shanghai, ranged in age from 50 to 81 years (M = 53.66 years, SD = 4.08 years), and 94.0% (*n* = 376) were aged 50–60 years. Approximately half of the respondents (51.3%, *n* = 205) were women, and a small majority had a bachelor’s degree (63.5%, *n* = 254). More than 71.0% of the respondents were full-time employed (*n* = 287). The monthly income of the majority of respondents fell within the range of 5001–8000 RMB (USD 692.01–1106.99, 27.8%, *n* = 111). Normal weight was the most prevalent condition (60.0%, *n* = 240), followed by overweight (25.0%, *n* = 100). Table A1 summarizes the sociodemographic analysis of Chinese respondents in Shanghai during the COVID-19 pandemic from July to August 2022 (see Appendix A).

The mean value of eating self-efficacy among respondents during the COVID-19 lockdown was high (M = 4.07, SD = 0.51), followed by health consciousness (M = 3.86, SD = 0.57), food literacy (M = 3.65, SD = 0.61), and negative emotions (M = 3.04, SD = 0.73). The paired results of the six main variables were significant, except for the correlation between eating self-efficacy and unhealthy eating habits (r = 0.065, *p* = 0.192). Table 1 shows the zero-order correlations of the main variables for Chinese participants residing in Shanghai during the COVID-19 lockdown in 2022.

### 4.2. Evaluation of Main Effects

The path analysis in this study was designed to explore the connection between media exposure and unhealthy eating habits among older individuals in Shanghai during the COVID-19 lockdown. The CMIN/DF (minimum discrepancy per degree of freedom) value of 3.641 is within the acceptable range, indicating the model’s discrepancy per degree of freedom is within reasonable limits. An RMSEA (root mean squared error approximation) value of 0.081 points to a fairly close fit of the model to the observed data, although values below 0.08 are typically seen as indicative of a good fit. The GFI (goodness-of-fit index) value stood at 0.999, reflecting a high goodness-of-fit and suggesting the model accurately represents the relationships among the variables. Both the IFI (incremental fit index) and CFI (comparative fit index) values were at 0.998, demonstrating excellent incremental fit and indicating that the model fits the data well in comparison to a baseline model.

**Figure 1:**
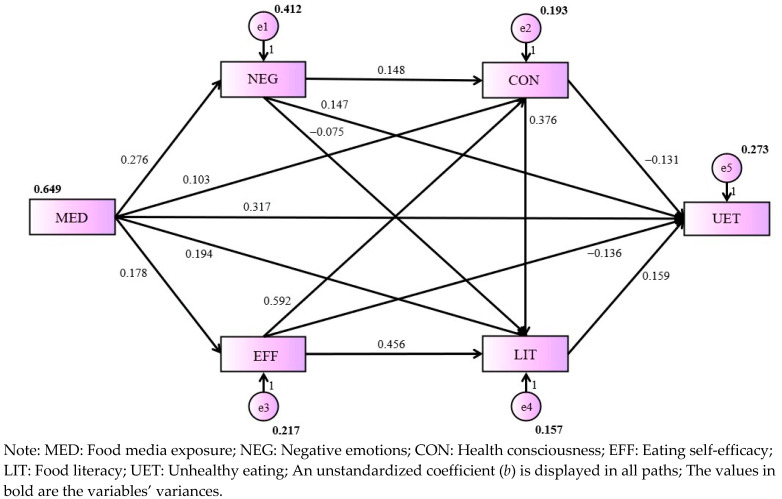
The path analysis model depicting the relationships among the six main observed variables (depicted as rectangles) and the five error terms (shown as circles), which collectively influence unhealthy eating habits among the older population in Shanghai.

Figure 1 presents the path-analysis diagram, illustrating the interactions between media exposure, negative emotions, health consciousness, food literacy, eating self-efficacy, and unhealthy eating habits among older individuals in Shanghai. Each arrow in the model symbolizes a hypothesized link between two variables, with its direction suggesting the assumed potential causal relationship. The path coefficients, represented next to each arrow, measure the degree to which variations in one variable are associated with changes in another, providing insight into the strength of these relationships.

How media exposure affects unhealthy eating habits is predicted. In the total-effects model for unhealthy eating, media exposure positively predicted unhealthy eating without controlling for negative emotions, health consciousness, food literacy, or eating self-efficacy (*b* = 0.357, 95% CI [0.275, 0.444], *p* < 0.001). Therefore, H1 is supported by these data.

H2 addressed the mediating role of negative emotions. The *p*-values for the relationships were statistically significant for both the effect of media exposure on negative emotions (*b* = 0.276, *p* < 0.001) and negative emotions on unhealthy eating (*b* = 0.147, *p* < 0.001), whereas negative emotions positively mediated the effect of food media exposure on unhealthy eating habits (*b* = 0.041, 95% CI [0.018, 0.076], *p* < 0.001). Thus, H2 is also supported.

H3 investigated the indirect effects of food literacy. Food media exposure was positively associated with food literacy (*b* = 0.194, *p* < 0.001). Food literacy positively predicted unhealthy eating habits (*b* = 0.159, *p* = 0.018). These two pathways showed that food literacy positively mediated the effect of media exposure on unhealthy eating (*b* = 0.031, 95% CI [0.002, 0.070], *p* = 0.036), thus supporting H3.

H4 tested the mediating role of health consciousness. Media exposure was positively correlated with health consciousness (*b* = 0.103, *p* = 0.003). Health consciousness was negatively associated with unhealthy eating habits (*b* = −0.131, *p* = 0.048). The product of these two coefficients was significant (*b* = −0.013, 95% CI [−0.038, −0.001], *p* = 0.036), which suggested that the negative mediating effect of health consciousness emerged in the process connecting media exposure with unhealthy eating. Hence, H4 is supported.

H5 investigated whether eating self-efficacy influenced the connection between media exposure and unhealthy eating. The effect of media exposure on eating self-efficacy was 0.178 (*p* < 0.001). Eating self-efficacy did not directly predict unhealthy eating (*b* = −0.136, *p* = 0.068) but mediated the path from media exposure through eating self-efficacy to unhealthy eating (*b* = −0.024, 95% CI [−0.059, 0.000], *p* = 0.047). Thus, H5 is supported.

Table 2 summarizes the six mediating pathways between media exposure and unhealthy eating habits in this model. Each row in the table outlines a specific relationship between two variables, with the direction indicating the suggested pathway. The strength of these relationships is estimated by the path unstandardized coefficients, which quantify the degree to which changes in one variable are associated with changes in another.

The RQ1 addressed how negative emotions, health consciousness, food literacy, and eating self-efficacy work together to influence the relationship between media exposure and unhealthy eating habits. Specifically, negative emotions positively predicted health consciousness (*b* = 0.148, *p* < 0.001), and health consciousness positively predicted food literacy (*b* = 0.376, *p* < 0.001). Combined with the effect of media exposure on negative emotions (*b* = 0.276, *p* = 0.003) and food literacy on unhealthy eating (*b* = 0.159, *p* = 0.018), these four paths showed that negative emotions, health consciousness, and food literacy positively mediated the relationship between media exposure and unhealthy eating (*b* = 0.002, 95% CI [0.000, 0.007], *p* = 0.027).

Additionally, eating self-efficacy positively predicted health consciousness (*b* = 0.592, *p* < 0.001). Combining this with the effect of media exposure on self-efficacy (*b* = 0.178, *p* < 0.001), the effect of health consciousness on food literacy (*b* = 0.376, *p* < 0.001), and the effect of food literacy on unhealthy eating (*b* = 0.159, *p* = 0.018), the combination of these four pathways was also significant (*b* = 0.006, 95% CI [0.000, 0.017], *p* = 0.035), showing that eating self-efficacy, health consciousness, and food literacy positively mediated the effect of media exposure on unhealthy eating.

**Figure 2:**
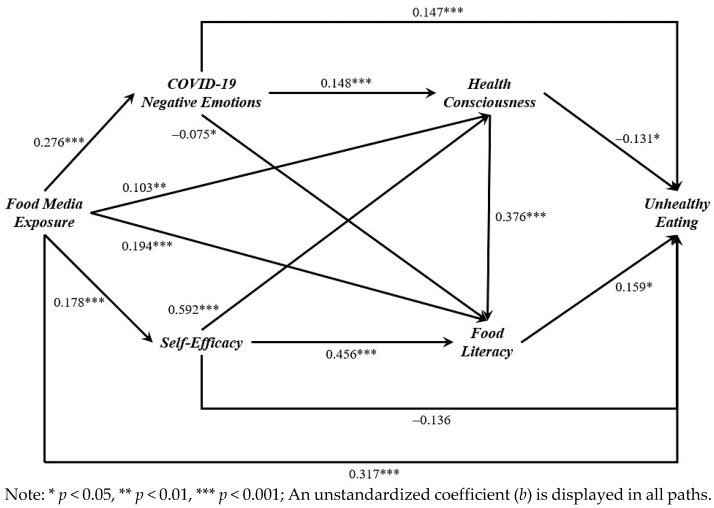
Statistical model of the cognitive process for unhealthy eating behavior among older Chinese adults in Shanghai during the COVID-19 lockdown in 2022.

Figure 2 shows the statistical model of the cognitive process underlying unhealthy eating behavior among older Chinese adults in Shanghai during the COVID-19 lockdown in 2022. It visualizes the relationships between four key factors. Each arrow in the diagram represents an identified relationship between two variables, along with its determined causal pathway. Path unstandardized coefficients that are significantly different demonstrate the extent to which changes in one variable influence changes in another, thereby indicating the strength of these relationships.

## 5. Discussion

### 5.1. Interpretation of Findings

This study contributes to a cognition model by examining how the factors of negative emotions, food literacy, health consciousness, and eating self-efficacy potentially mediated the connection between media exposure and unhealthy eating behaviors among older Chinese individuals during the COVID-19 lockdown in Shanghai. The path analysis offers valuable insights into the intricate dynamics between media exposure and unhealthy eating habits among older individuals in Shanghai during the COVID-19 lockdown. The model’s satisfactory fit statistics suggest that it effectively captures the interactions among the variables, emphasizing the need to consider various factors in understanding and addressing unhealthy eating behaviors in this demographic.

First, this study applied the cognition mechanism to a new context—the COVID-19 pandemic lockdown. The effect of media exposure on unhealthy eating is particularly pronounced in older adults. They are inclined to consume unhealthy foods through observational learning and modeling triggered by media content [54], which supports previous findings that highlight the increased health burden of the current food media environment [55,56]. This study may also provide fresh perspectives and insights into the cognition mechanism of other relevant public health emergencies and their potential influences.

Second, using the variables of negative emotions, food literacy, health consciousness, and self-efficacy, this study helps us understand how the media shapes public perceptions of unhealthy eating and how these variables interact in potentially socially mediated pathways [54]. The result shows that negative emotions represented the largest share of mediators and played a significant role in intensifying the impact of food media on unhealthy eating habits. This is consistent with findings of previous studies showing that negative emotions exacerbated overeating and cravings for energy-dense, flavorful, unhealthy foods during a COVID-19 lockdown [5,18,19]. Food media exposure can lead to heightened negative emotions resulting from uncontrollable, unhealthy binge eating, which can, in turn, exacerbate both general and mental health risks in older people [21].

Third, the mediating effect of food literacy was significantly positive in shaping the relationship between food media exposure and unhealthy eating among older adults, accounting for the second-largest proportion of the total effect. This is contrary to the common belief that food literacy improves healthy eating behaviors because food-related content is an effective tool [24,25]. Older individuals with greater food literacy may still consume more unhealthy foods than their younger counterparts. Food literacy has a dual effect on healthy and unhealthy eating behaviors.

Fourth, health consciousness mitigated the catalytic effect of food media exposure on unhealthy eating habits among older adults. This is supported by studies that indicate the importance of food media in raising public consciousness [27,28] and the negative relationship between health consciousness and the consumption of unhealthy foods [31]. Evidence from this diet-protection route can bolster older people’s reasoning during an ongoing public health emergency; when they are exposed to stimuli that potentially highlight health risks, the corresponding functions of rational cognition and thinking are activated [36,57].

Fifth, the mediating effect of eating self-efficacy alone was the third largest in the process linking food media exposure to unhealthy eating. These findings support the idea that self-efficacy can effectively control unhealthy behaviors, as demonstrated in previous studies [33,34]. Similar to health consciousness, eating self-efficacy inhibited the catalytic effect of food media exposure on unhealthy eating habits. Older generations may perceive that they possess greater autonomy in managing their health and exhibit a higher level of assurance regarding their dietary choices [57]. These findings echo the focal role of self-efficacy in directly and indirectly predicting and ameliorating changes in eating behavior.

Sixth, two renewal parallel mechanisms were observed in the relationship between food media exposure and unhealthy eating: one pathway is from negative emotions to health consciousness and then to food literacy, and the other is from eating self-efficacy to health consciousness and then to food literacy. Both serve as accelerators of the media’s influence on unhealthy eating habits. This cognitive-behavioral approach has expanded on a socially mediated pathway [54]; media stimuli trigger constructive individual behavior through sequential increases in psychosocial factors. However, the percentages of these two pathways are negligible, and they act as effect modifiers in the overall cognitive process [58]. Furthermore, this demonstrates that it might be advantageous and practical to examine the effects of individual factors on mitigating the consequences of food media exposure on unhealthy eating habits.

### 5.2. Practical Implications

This study has important practical implications for enhancing the dietary health of older Chinese adults and offers a novel perspective on the establishment of life-course prevention strategies for public health emergencies. First, considering the negative effects of exposure, it is the responsibility of food media to disseminate more information about healthy eating patterns, such as increasing public awareness of these topics and encouraging people to make healthier dietary choices rather than being overwhelmed by unhealthy food messages and advertisements. Second, the content of food media broadcasts should reduce the likelihood of increased negativity. When negative emotions trigger emotional eating, older adults can use stress management techniques to control the urge for comfort foods. Third, increasing health consciousness and eating self-efficacy are powerful ways to counteract the accelerating effects of food media exposure on unhealthy eating habits. It is timely for the relevant authorities and policymakers to emphasize to older adults the importance of personal initiatives in health promotion and self-regulation.

### 5.3. Limitations and Future Directions

This study has some limitations. First, an overwhelming percentage of analyzed respondents were aged 50–60 years, and this study considers the effect of age as a total vector. Future studies should expand the sample size to collect more data. Second, this study considered that people are exposed to food-related information from various media sources; therefore, the effect of food media exposure is significant only as a composite and total vector. Indeed, active media-seeking behavior differs substantially from passive media consumption. Consequently, active search behavior could be measured as a separate variable in future research, and a comparison of the effects of mass and social media could be considered. Third, this was a cross-sectional study using panel data at a single point in time. By identifying potential correlations, associations, and relationships between variables, this study assessed the degree to which the datasets corresponded to the proposed model suppositions; however, this does not confirm causality. Experimental methods could be applied in future research to measure these changes and confirm the occurrence of pre- and post-change variables.

## 6. Conclusions

This study demonstrated the role of media on the cognitive processes related to unhealthy eating among older adults in Shanghai, China, during the COVID-19 lockdown. It highlights the deep interplays between negative emotions, eating self-efficacy, health consciousness, and food literacy, all of which are influenced by food media exposure. In other words, this study expands upon prior research by expanding a relatively stable, verifiable, and nearly saturated theoretical framework to the field of cognitive behavior. This study also contributes to future research to better understand the complex interconnections between psychosocial factors and behavioral patterns in aging research. Additionally, this study has practical significance for media professionals, public health decision-makers, and healthy food businesses because its findings offer insights into how older people’s cognition of how to respond to unhealthy eating crises may be enhanced. It addresses the social concerns and provides policymakers with reference recommendations for interventions to improve eating habits and reduce social healthcare costs.

## Figures and Tables

**Table 1:** Zero-order correlation of the main variables among Chinese respondents in Shanghai during the COVID-19 lockdown in 2022.

Variable	Mean	SD	2	3	4	5	6
1: Food media exposure	2.95	0.81	0.371 **	0.460 **	0.321 **	0.279 **	0.522 **
2: COVID-19 negative emotions	3.04	0.73		0.187 **	0.313 **	0.153 **	0.316 **
3: Food literacy	3.65	0.61			0.631 **	0.645 **	0.239 **
4: Health consciousness	3.86	0.57				0.581 **	0.107 *
5: Eating self-efficacy	4.07	0.51					0.065
6: Unhealthy eating	2.60	0.64					

Note: ** *p* < 0.01; * *p* < 0.05. COVID-19, coronavirus disease 2019; SD, standard deviation.

**Table 2:** Mediation paths between food media exposure and unhealthy eating among older Shanghainese during the COVID-19 lockdown.

Parameter	Estimate	95% CI		*p*-Value
		Lower	Upper	
1: Food media exposure → Negative emotions → Unhealthy eating	0.041	0.018	0.076	<0.001
2: Food media exposure → Food literacy → Unhealthy eating	0.031	0.002	0.070	0.036
3: Food media exposure → Health consciousness → Unhealthy eating	−0.013	−0.038	−0.001	0.036
4: Food media exposure → Emotional eating → Unhealthy eating	−0.024	−0.059	0.000	0.047
5: Food media exposure → Negative emotions → Health consciousness → Food literacy → Unhealthy eating	0.002	0.000	0.007	0.027
6: Food media exposure → Eating self-efficacy → Health consciousness → Food literacy → Unhealthy eating	0.006	0.000	0.017	0.035

Note: The covariates include sex, education, employment status, income, subjective social status, general financial struggles, financial struggles for food, and body mass index. CI, confidence interval.

## Data Availability

The original contributions presented in the study are included in the article/supplementary material, further inquiries can be directed to the corresponding author.

## Supplementary Materials

The following supporting information can be downloaded at: https://www.mdpi.com/article/10.3390/foods13121797/s1, Table S1: Detailed measurements and scales of variables.

## Appendix A

**Table A1:** Sociodemographic analysis of Chinese respondents in Shanghai during the COVID-19 pandemic from July to August 2022.

Variable	Frequency	Percent
Gender		
Female	205	51.3%
Male	195	48.8%
Age		
50–60	376	94.0%
61–70	22	5.5%
71–80	1	0.3%
81–90	1	0.3%
Highest education		
Under a high school diploma	8	2.0%
High school diploma	88	22.0%
Bachelor’s degree	254	63.5%
Master’s degree	39	9.8%
Doctorate	11	2.8%
Employment status		
No work	55	13.8%
Work less than half-time	13	3.3%
Work half-time	23	5.8%
Work more than half-time but not full-time	22	5.5%
Work full time	287	71.8%
Monthly income in RMB		
1500 and below (≤USD 207.56)	1	0.3%
1501–2000 (USD 207.70–276.75)	0	0.0%
2001–3000 (USD 276.89–415.12)	3	0.8%
3001–5000 (USD 415.26–691.87)	32	8.0%
5001–8000 (USD 692.01–1106.99)	111	27.8%
8001–12,000 (USD 1107.13–1660.49)	102	25.5%
12,001–20,000 (USD 1660.63–2767.48)	87	21.8%
More than 20,000 (>USD 2767.48)	64	16.0%
Income changed due to COVID-19		
A lot less	14	3.5%
A little less	141	35.3%
No change	219	54.8%
A little more	22	5.5%
A lot more	4	1.0%
Body mass index		
Underweight	44	11.0%
Normal weight	240	60.0%
Overweight	100	25.0%
Obesity	16	4.0%

## References

[1] Hall B.J., Li G., Chen W., Shelley D., Tang W. (2023). Prevalence of depression, anxiety, and suicidal ideation during the Shanghai 2022 Lockdown: A cross-sectional study. J. Affect. Disord..

[2] Di Renzo L., Gualtieri P., Pivari F., Soldati L., Attinà A., Cinelli G., Leggeri C., Caparello G., Barrea L., Scerbo F. (2020). Eating habits and lifestyle changes during COVID-19 lockdown: An Italian survey. J. Transl. Med..

[3] Chang A., Schulz P.J., Jiao W., Liu M.T. (2021). Obesity-related communication in digital Chinese news from mainland China, Hong Kong, and Taiwan: Automated content analysis. JMIR Public Health Surveill..

[4] Bonaccio M., Costanzo S., Bracone F., Gialluisi A., Di Castelnuovo A., Ruggiero E., Esposito S., Olivieri M., Persichillo M., Cerletti C. (2022). Psychological distress resulting from the COVID-19 confinement is associated with unhealthy dietary changes in two Italian population-based cohorts. Eur. J. Nutr..

[5] Jiao W., Xiang Y.T., Chang A. (2022). Are foods from the COVID-19 pandemic lockdown low in nutrients? An analysis of Chinese psychological distress effects. Nutrients.

[6] Bakaloudi D.R., Jeyakumar D.T., Jayawardena R., Chourdakis M. (2022). The impact of COVID-19 lockdown on snacking habits, fast-food and alcohol consumption: A systematic review of the evidence. Clin. Nutr..

[7] Sidor A., Rzymski P. (2020). Dietary choices and habits during COVID-19 lockdown: Experience from Poland. Nutrients.

[8] Elisabeth A.L., Karlen S.B., Magkos F. (2021). The effect of COVID-19-related lockdowns on diet and physical activity in older adults: A systematic review. Aging Dis..

[9] Paderno A., Schreiber A., Grammatica A., Raffetti E., Tomasoni M., Gualtieri T., Taboni S., Zorzi S., Lombardi D., Deganello A. (2020). Smell and taste alterations in COVID-19: A cross-sectional analysis of different cohorts. Int. Forum Allergy Rhinol..

[10] Chen Y., Klein S.L., Garibaldi B.T., Li H., Wu C., Osevala N.M., Li T., Margolick J.B., Pawelec G., Leng S.X. (2021). Aging in COVID-19: Vulnerability, immunity and intervention. Ageing Res. Rev..

[11] Lu G., Zhang Y., Zhang H., Ai J., He L., Yuan X., Bao S., Chen X., Wang H., Cai J. (2022). Geriatric risk and protective factors for serious COVID-19 outcomes among older adults in Shanghai Omicron wave. Emerg. Microbes Infect..

[12] Decorte P., Cuykx I., Teunissen L., Poels K., Smits T., Pabian S., van Royen K., De Backer C. (2022). “Everywhere you look, you’ll find food”: Emerging adult perspectives toward the food media landscape. Ecol. Food Nutr..

[13] Renström E.A., Bäck H. (2021). Emotions during the COVID-19 pandemic: Fear, anxiety, and anger as mediators between threats and policy support and political actions. J. Appl. Soc. Psychol..

[14] Ahorsu D.K., Lin C.Y., Imani V., Saffari M., Griffiths M.D., Pakpour A.H. (2022). The Fear of COVID-19 Scale: Development and initial validation. Int. J. Ment. Health Addict..

[15] Sim S., Wiwanitkit V. (2021). Food contamination, food safety and COVID-19 outbreak. J. Health Res..

[16] Kaneko D., Toet A., Brouwer A.M., Kallen V., Van Erp J.B.F. (2018). Methods for evaluating emotions evoked by food experiences: A literature review. Front. Psychol..

[17] Naja F., Hamadeh R. (2020). Nutrition amid the COVID-19 pandemic: A multi-level framework for action. Eur. J. Clin. Nutr..

[18] Zhang Y.T., Li R.T., Sun X.J., Peng M., Li X. (2021). Social media exposure, psychological distress, emotion regulation, and depression during the COVID-19 outbreak in community samples in China. Front. Psychiatry.

[19] McAtamney K., Mantzios M., Egan H., Wallis D.J. (2021). Emotional eating during COVID-19 in the United Kingdom: Exploring the roles of alexithymia and emotion dysregulation. Appetite.

[20] Camilleri G.M., Méjean C., Kesse-Guyot E., Andreeva V.A., Bellisle F., Hercberg S., Péneau S. (2014). The associations between emotional eating and consumption of energy-dense snack foods are modified by sex and depressive symptomatology. J. Nutr..

[21] Lopez-Cepero A., Frisard C.F., Lemon S.C., Rosal M.C. (2019). Association between emotional eating, energy-dense foods and overeating in Latinos. Eat. Behav..

[22] Vidgen H.A., Gallegos D. (2014). Defining food literacy and its components. Appetite.

[23] Poelman M.P., Dijkstra S.C., Sponselee H., Kamphuis C.B.M., Battjes-Fries M.C.E., Gillebaart M., Seidell J.C. (2018). Towards the measurement of food literacy with respect to healthy eating: The development and validation of the self perceived food literacy scale among an adult sample in the Netherlands. Int. J. Behav. Nutr. Phys. Act..

[24] Steils N., Obaidalahe Z. (2020). “Social food”: Food literacy co-construction and distortion on social media. Food Policy.

[25] Melki J., Tamim H., Hadid D., Farhat S., Makki M., Ghandour L., Hitti E. (2022). Media exposure and health behavior during pandemics: The mediating effect of perceived knowledge and fear on compliance with COVID-19 prevention measures. Health Commun..

[26] Hong H. (2011). An extension of the extended parallel process model (EPPM) in television health news: The influence of health consciousness on individual message processing and acceptance. Health Commun..

[27] Lapointe L., Ramaprasad J., Vedel I. (2014). Creating health awareness: A social media enabled collaboration. Health Technol..

[28] Igbinoba A.O., Soola E.O., Omojola O., Odukoya J., Adekeye O., Salau O.P. (2020). Women’s mass media exposure and maternal health awareness in Ota, Nigeria. Cogent Soc. Sci..

[29] Dutta M.J. (2007). Health information processing from television: The role of health orientation. Health Commun..

[30] Divine R.L., Lepisto L. (2005). Analysis of the healthy lifestyle consumer. J. Consum. Mark..

[31] Hartmann C., Siegrist M., van der Horst K. (2013). Snack frequency: Associations with healthy and unhealthy food choices. Public Health Nutr..

[32] Jiao W., Liu M.T., Schulz P.J., Chang A. (2022). Impacts of self-efficacy on food and dietary choices during the first COVID-19 lockdown in China. Foods.

[33] Fitzgerald A., Heary C., Kelly C., Nixon E., Shevlin M. (2013). Self-efficacy for healthy eating and peer support for unhealthy eating are associated with adolescents’ food intake patterns. Appetite.

[34] Churchill S., Good A., Pavey L. (2014). Promoting the avoidance of high-calorie snacks. The role of temporal message framing and eating self-efficacy. Appetite.

[35] Diotaiuti P., Valente G., Mancone S., Corrado S., Bellizzi F., Falese L., Langiano E., Vilarino G.T., Andrade A. (2023). Effects of cognitive appraisals on perceived self-efficacy and distress during the COVID-19 lockdown: An empirical analysis based on structural equation modeling. Int. J. Environ. Res. Public Health.

[36] Van Kampen H.S. (2019). The principle of consistency and the cause and function of behaviour. Behav. Process..

[37] Hoque M.Z., Alam M.N., Nahid K.A. (2018). Health consciousness and its effect on perceived knowledge, and belief in the purchase intent of liquid milk: Consumer insights from an emerging market. Foods.

[38] Anderson E.S., Winett R.A., Wojcik J.R. (2000). Social-cognitive determinants of nutrition behavior among supermarket food shoppers: A structural equation analysis. Health Psychol..

[39] Mai R., Hoffmann S. (2012). Taste lovers versus nutrition fact seekers: How health consciousness and self-efficacy determine the way consumers choose food products. J. Consum. Behav..

[40] Rimal R.N. (2000). Closing the knowledge-behavior gap in health promotion: The mediating role of self-efficacy. Health Commun..

[41] Cha E., Kim K.H., Lerner H.M., Dawkins C.R., Bello M.K., Umpierrez G., Dunbar S.B. (2014). Health literacy, self-efficacy, food label use, and diet in young adults. Am. J. Health Behav..

[42] De Backer C., Teunissen L., Cuykx I., Decorte P., Pabian S., Gerritsen S., Matthys C., Al Sabbah H., Van Royen K., Corona Cooking Survey Study Group (2020). An evaluation of the COVID-19 pandemic and perceived social distancing policies in relation to planning, selecting, and preparing healthy meals: An observational study in 38 countries worldwide. Front. Nutr..

[43] Grunert K.G., Janssen M., Nyland Christensen R., Teunissen L., Cuykx I., Decorte P., Reisch L.A. (2022). “Corona Cooking”: The interrelation between emotional response to the first lockdown during the COVID-19 pandemic and cooking attitudes and behaviour in Denmark. Food Qual. Prefer..

[44] Zhang X., Zhang W., Chen S. (2022). Shanghai’s life-saving efforts against the current omicron wave of the COVID-19 pandemic. Lancet.

[45] Giles J., Lei X., Wang G., Wang Y., Zhao Y. (2023). One country, two systems: Evidence on retirement patterns in China. J. Pension Econ. Financ..

[46] Mottram S., Peat G., Thomas E., Wilkie R., Croft P. (2008). Patterns of pain and mobility limitation in older people: Cross-sectional findings from a population survey of 18,497 adults aged 50 years and over. Qual. Life Res..

[47] Ma J.F., Xin X.Y., Liang L., Liu L.H., Fang R., Zhang Y.J., Wang D.Y., Fahn S., Tang H.D., Chen S.D. (2012). Restless legs syndrome in Chinese elderly people of an urban suburb in Shanghai: A community-based survey. Parkinsonism Relat. Disord..

[48] Yang F., Gu D., Mitnitski A. (2016). Frailty and life satisfaction in Shanghai older adults: The roles of age and social vulnerability. Arch. Gerontol. Geriatr..

[49] Xiao Y., Miao S., Zhang Y., Xie B., Wu W. (2022). Exploring the associations between neighborhood greenness and level of physical activity of older adults in shanghai. J. Transp. Health.

[50] Glynn S.M., Ruderman A.J. (1986). The development and validation of an Eating Self-Efficacy Scale. Cogn. Ther. Res..

[51] Weihrauch A., Huang S.C. (2021). Portraying humans as machines to promote health: Unintended risks, mechanisms, and solutions. J. Mark..

[52] Williams J., Mackinnon D.P. (2008). Resampling and distribution of the product methods for testing indirect effects in complex models. Struct. Equ. Modeling.

[53] Schulz P.J., Fitzpatrick M.A., Hess A., Sudbury-Riley L., Hartung U. (2017). Effects of eHealth literacy on general practitioner consultations: A mediation analysis. J. Med. Internet Res..

[54] Bandura A. (2004). Health promotion by social cognitive means. Health Educ. Behav..

[55] Jiao W., Chang A. (2020). Unhealthy aging? Featuring older people in television food commercials in China. Int. J. Nurs. Sci..

[56] Parvanta S.A., Brown J.D., Du S., Zimmer C.R., Zhao X., Zhai F. (2010). Television use and snacking behaviors among children and adolescents in China. J. Adolesc. Health.

[57] Jiao W., Chang A., Ho M., Lu Q., Liu M.T., Schulz P.J. (2023). Predicting and empowering health for generation Z by comparing health information seeking and digital health literacy: Cross-sectional questionnaire study. J. Med. Internet Res..

[58] Bours M.J.L. (2023). Using mediators to understand effect modification and interaction. J. Clin. Epidemiol..

