# Supplementary material for: Older Adults’ Exposure to Food Media Induced Unhealthy Eating during the COVID-19 Omicron Lockdown? Exploring Negative Emotions and Associated Literacy and Efficacy on Shanghainese
Source: Foods. 2024 Jun 7;13(12):1797. doi: 10.3390/foods13121797 (PMC11203190; doi:10.3390/foods13121797)
Supplement: Supplementary file 1 [file foods-13-01797-s001.zip › foods-3035026-supplementary.pdf]

Supplementary Materials

Table S1. Detailed information about measurements.

Food Media Exposure (1 = Never; 5 = All the time)

During the COVID-19 lockdown, how often have you received messages about food and nutrition from each of the media listed?

- Cookbooks
- Other print media (e.g., magazines, newspapers, recipe cards)
- Sina Weibo
- TV shows (including iQIYI, Tencent video, Youku video)
- Facebook
- Forum websites (e.g., Tianya, douban)
- Instagram
- WeChat
- YouTube
- Tik Tok
- bilibili
- Other social media

Unhealthy Eating (1 = Less than a few times per month or never; 5 = More or many times a day)

During the COVID-19 lockdown, how often do you take in the following foods and drinks?

- Processed meat
- Red meat
- Sweet snacks
- Salty snacks
- Sweetened beverages
- Alcoholic beverages
- Fast food
- Animal fats
- Coconut oil
- White grains

Negative Emotions Associated With COVID-19 (1 = Strongly disagree; 5 = Strongly agree)

Psychological Distress

During the COVID-19 lockdown, how often have you felt?

- hopeless
- restless or fidgety
- worthless
- nervous
- that everything requires effort
- so depressed that nothing could cheer you up

Fear of COVID-19

During the COVID-19 lockdown, how often have you felt?

- I am most afraid of COVID-19
- It makes me uncomfortable to think about COVID-19
- My hands become clammy when I think about COVID-19
- I am afraid of losing my life because of COVID-19
- When watching news and stories about COVID-19 on social media, I become nervous or anxious
- I cannot sleep because I'm worrying about getting COVID-19
- My heart races or palpitates when I think about getting COVID-19

Food Literacy (1 = Never; 5 = Every time)

During the COVID-19 lockdown, to what extent have you taken the following food actions?

- Plan meals ahead of time
- Make a list before you go shopping
- Try a new recipe
- Plan meals to include all food groups
- Think about healthy choices when deciding what to eat
- Feel confident about managing money to buy healthy food
- Use the nutritional information panel (nutritional breakdown of the products) to make food choices
- Use other parts of food label to make food choices (such as which ingredients a product contains)
- Cook meals at home using healthy ingredients
- Feel confident about cooking a variety of healthy meals
- Change recipes to make them healthier

Health Consciousness (1 = Never; 5 = All the time)

During the COVID-19 lockdown, how often have you been thinking of the health subjects listed?

- Reflecting on my health
- Being aware of my health
- Examining the state of my health
- Being alert to changes in my health
- Feeling overly self-conscious about my health
- Attending to my inner feelings about my health in general
- Being aware of the state of my health as I go through the day
- Noticing how I feel physically as I go through the day
- Involving myself with my health

Eating Self-Efficacy (1 = Strongly disagree; 5 = Strongly agree)

To what extent do you agree with the following statements?

- I believe I have the ability to eat healthy food
- I can eat healthily
- I have confidence in the healthy diet lifestyle
- If it's entirely up to me, I believe I can have a healthy diet
- I can maintain a healthy diet when I'm busy or in a hurry
- I know what kind of food I should eat
- I can add vegetables and other foods rich in dietary fiber to my eating
- I know how to cook healthy food
- When I eat alone, I can prepare healthy food for myself
- When shopping, I know that buying food is healthy
- I can achieve my ideal weight through a healthy diet

Sociodemographic Variables

Gender

- Female
- Male

Age (Ranging from 50 to 120 years old)

Height (Ranging from 100 to 230 centimeters)

Weight (Ranging from 40 to 200 kilograms)

Education

- Under a high school diploma (or none)
- High school diploma or equivalent
- Bachelor's degree or equivalent
- Master's degree or equivalent
- Doctorate

Income

What's your monthly income (RMB)?

- 1500 and below
- 1501–2000
- 2001–3000
- 3001–5000
- 5001–8000
- 8001–12,000
- 12,001–20,000
- More than 20,000

During the COVID-19 lockdown, how has your income changed?

- A lot less
- A little less
- Did not change
- A little more
- A lot more

Employment Status

- I currently do not work (student not working, unemployed, retired, other)
- I work less than half-time
- I work half-time (50%)
- I work more than half-time, but not full-time
- I work full-time (or more)

General Financial Struggles (1 = Never; 7 = Every time)

In general, how often is it a struggle to make your money last until the end of the month/payday?

Financial Struggles for Food (1 = Never; 7 = Every time I go shopping for food)

In general, how often is it a struggle to have enough money to go shopping for food?
